# Sample size determination for time-to-event endpoints in randomized selection trials with generalized exponential distribution

**DOI:** 10.1016/j.heliyon.2024.e27013

**Published:** 2024-02-28

**Authors:** Muhammad Hamza Akbar, Sajid Ali, Ismail Shah, Hana N. Alqifari

**Affiliations:** a Department of Statistics, Quaid-i-Azam University, Islamabad 45320, Pakistan; b Department of Statistical Sciences, University of Padua, 35121, Padova, Italy; c Department of Statistics and Operation Research, College of Science, Qassim University, Buraydah, 51482, Saudi Arabia

**Keywords:** Generalized exponential distribution, Randomized control trials, Sample size determination, Weibull distribution

## Abstract

Randomized selection trials are frequently used to compare experimental treatments that have the potential to be beneficial, but they often do not include a control group. While time-to-event endpoints are commonly applied in clinical investigations, methodologies for determining the required sample size for such endpoints, except exponential distribution, are lacking. In recent times, there has been a shift in clinical trials, with a growing emphasis on progression-free survival as a primary endpoint. However, the utilization of this measure has typically been restricted to specific time points for both sample size determination and analysis. This alteration in approach could wield a substantial influence on the clinical trial process, potentially diminishing the capacity to discern variances between treatment groups. In the calculation of sample sizes for randomized trials, this investigation operates under the assumption that the time-to-event endpoint conforms to either an exponential, Weibull, or generalized exponential distribution.

## Highlights

• Sample size calculation for time-to-event endpoints is discussed using a generalized exponential distribution.
• Comparing results with Weibull distribution, a smaller sample size is achieved with the proposal.
• **Problem:** Time-to-event endpoints may not follow well-known distributions like exponential or Weibull.
• **What is Already Known:** Computing sample size using Weibull distribution ignored previously the ineffectiveness of the maximum likelihood estimation.
• **What This Paper Adds:** This study proposed a sample size calculation based on a generalized exponential distribution to achieve the desired power.

## 1 Introduction

Clinical trials are research studies that test the effectiveness of medical, surgical, or behavioral interventions on humans. Most researchers rely on these trials to evaluate whether new therapies or interventions, such as a new medication, diet, or medical device (such as a pacemaker), are safe and effective in humans. In the majority of cases, clinical trials are designed to test whether a new treatment is more efficient or has fewer side effects than the existing treatment [1]. It also aims to diagnose a disease early sometimes before there are any symptoms and also improve the quality of life for people suffering from life-threatening diseases. Thus, to fulfill these aims people volunteer for clinical trials. The participants of a trial are usually referred to as clinical subjects. The number of participants in a trial, thus, becomes a major part of the process.

The calculation of sample size (SS) to achieve the research objectives is a key step in the design of clinical studies and it must be chosen after careful consideration [2]. Planning the SS is essential if the population to be investigated for the study is challenging to examine, such as a population with a rare condition where there are enough participants to undertake a trial of the standard size or a situation where trial participation is constrained, such as pediatric populations. The amount of precision needed, the anticipated impact size, the outcome's unpredictability, and the desired level of significance all play a role in determining SS. In terms of statistical power, the SS should be sufficient to distinguish the experimental group from the control group without being excessive in terms of resource consumption. Additionally, false negative results may be caused by insufficient sample sizes, whereas false positive results may be caused by excessive sample sizes.

The design and analysis of randomized clinical trials focus largely on the expected time until an event occurs as the key outcome variable. The key outcome variable also known as the time-to-event outcome variable reflects the time until a participant has an event of interest. Sample size calculation methods are well-established for binary endpoints, but for time-to-event endpoints, which are commonly utilized in clinical trials, there is a lack of corresponding methods except the exponential distribution as discussed by Kieser [3]. Schober and Vetter [4] pointed out that the event under consideration should have clinical significance, a clear and precise definition, lack any ambiguity, and ideally, be easily observable.

Lewis [5] discussed that the SS is dependent upon the trial's characteristics. For example, the therapy for many chronic diseases seeks to stop or lessen instances of a disease while for a rare chronic disease, the situation might be different. In such scenarios, entry criteria for clinical trials commonly involve estimating a minimum number of occurrences within a baseline period. McMahon et al. [6] gave SS formulae that take account of the entry criterion, and derived them for comparison of the mean number of events at follow-up and the proportion of patients with zero events at follow-up. Sargent and Goldberg [7] discussed a design guided by a specific decision criterion. Gogtay [8] discussed various formulae to calculate the SS. Chow [9] discussed three approaches for SS calculation which can be employed depending on the situation. The typical three distinct methods rely on the analysis of precision, analysis of power, and formulation of probability statements. Precision analysis is used to control the rate of Type I error. Power analysis is designed to attain a targeted likelihood of accurately identifying a clinically significant distinction when such a distinction genuinely exists. The intent behind the probability statement is to ensure that the chance of observing a specific event is lower than a predetermined set of values.

Iasonos and O'Quigley [10] discussed the role of randomization in the early phases of a clinical trial. Pourhoseingholi et al. [11] discussed certain suggestions for distinct phases within a clinical trial stemming from considerations of SS. Spiegelhalter and Freedman [12] identified the weakness in the ‘textbook’ approach for the calculation of SS. To overcome that weakness a new prediction technique is introduced that considers previous clinical opinions regarding the treatment difference. Cotterill and Whitehead [13] discussed Phase II trials involving binary endpoints for time-to-event outcomes. Bayesian computations for SS are outlined for both single-arm and randomized Phase II studies, incorporating proportional hazard models as the basis for time-to-event endpoints. Later Kakizume et al. [14] extended this idea further in explanatory trials. Zhou et al. [15] discussed a Bayesian optimal Phase II design tailored for time-to-event endpoints by employing exponential-inverse gamma model.

Dehbi and Hackshaw [16] computed sample sizes for both two- and three-arm randomized selection trials using precise binomial probabilities, with a predefined margin of practical equivalence (MPE). Later, Dehbi et al. [17] developed the theory for SS calculation in selection trials. Billings et al. [18] gave an alternative approach for the calculation of SS. This approach is based on the assumption that both the Type I and Type II errors were symmetric. This approach is heavily grounded in the assumption that the mean treatment response among groups is well-established, enabling precise medical decision-making and consequently minimizing the likelihood of a Type III error. The emphasis of this approach is on optimality, rendering the role of the powerless pertinent. Miller et al. [19] discussed different approaches for SS calculation for clinical trials in rare diseases. Wang and Chow [20] discussed the significance of time-to-event within clinical trials. Assumptions revolving around proportional hazards (PH) or the exponential distribution of survival times have been conventionally integrated into SS estimations for two-arm clinical trials featuring a time-to-event endpoint. Phadnis and Mayo [21] discussed a methodology for calculating sample sizes in specific cases of both non-proportional hazard and non-proportional time scenarios has been developed. This involves considering that the survival durations for the control and treatment arms stem from two distinct Weibull distributions with varying location and shape parameters.

The conventional objective of power for a significance test may not always be an achievable or desirable goal for the investigation. However, selecting this approach and then specifying the necessary parameters for it fairly is a crucial stage in determining SS. This needs to be done so that the SS may neither exceed nor fall short of the target value. Wrong choice of parameter may lead to either a very large or small value of the SS which has serious consequences in the trial. If the SS is too small, it becomes difficult to detect a relevant treatment effect. However, if it is too large, patients enrolled in the trial later might be assigned to a treatment that is known to be less effective [3].

This study involves a selection trial [17] with time-to-event endpoints and the main objective of this research is to determine SS assuming generalized exponential distribution and further compare it with Weibull distribution based calculation. This study is useful to determine how well the drug works in subjects at a given dose to assess efficacy [22], [23], [24], [25]. The reason for considering this particular distribution is explained as follows. According to Gupta and Kundu [26], three-parameter survival distributions like gamma, Weibull, exponentiated Weibull distribution, etc., contain three parameters namely location, shape, and scale, which contribute toward their adaptability and suitability for different domains of life. These are preferable to use than the exponential distribution which has a constant hazard rate. In these distributions, the hazard rate changes as the shape parameter changes as it is not constant like exponential distribution. However, there are certain limitations associated with the mentioned distributions. For instance, in the context of the Weibull distribution, the maximum likelihood estimation (MLE) becomes ineffective for specific values of the location parameter. Similarly, the median survival for the gamma distribution is obtained numerically. Thus, an alternative probability distribution, known as the generalized exponential distribution, is introduced by Gupta and Kundu [27], which has a closed-form hazard function and competing model to gamma and Weibull models. This distribution can be seen as a specific instance of the exponentiated Weibull distribution when the location parameter is set to zero. The discrimination between the Weibull and generalized exponential distributions is thoroughly explained in a study by Gupta and Kundu [28].

The rest of the study is divided into four different sections. In Section 2, methods that are used for the analysis of SS calculation are presented. The results are discussed in Section 3. The conclusion and recommendations are given in Section 4.

## 2 Methodology for SS calculation for different distributions

This section discusses different methods for the calculation of SS for data generated from two different distributions. Let the dosage levels be selected and the trial would end in any of three possible states: superiority, practical equivalence, and inferiority. Let $p_{sup},p_{inf},p_{equi}$ denote the probabilities associated with these three states and $p_{sup}+p_{inf}+p_{equi}=1$. The probability of the efficacious dose selected is defined as *π*. This may occur in one of two ways.

(1) If the observed median for dose 2 surpasses that of dose 1's median by an extent greater than the MPE, which occurs with probability $p_{sup}$.
(2) The observed medians lie within the MPE range, which happens with probability $p_{equi}$. Therefore, the selection of dose 2 is determined by other factors, such as toxicity and quality of life.

The principle of indifference is applied in the case of lack of information about dose 2 and $\pi =p_{sup}+0.5p_{equi}$. Thus, it is crucial to calculate SS given a hypothesized median and MPE for the given two doses which ensures that the *π* exceeded some threshold value, i.e., 0.80. Such studies are used to determine how well the drug works in subjects at a given dose to assess efficacy [3], [22], [23], [24], [25].

### 2.1 SS for exponentially distributed randomized trial

The reason for considering exponential distribution is its simplicity and wide application in time-to-event studies. Assuming no censoring, consider a trial that compares two levels of different dosages, specifically labeled as 1 and 2. The survival times, denoted as *T* follow an exponential distribution, i.e., $T_{1}∼Exp(\theta_{1}$) and $T_{2}∼Exp(\theta_{2}$), $\theta_{i}>0$. The exponential density function is given by:

$$g(t)=\theta e^{-\theta t},\;t>0$$

Assuming $\theta_{1}>\theta_{2}$, that the median time for dose 1 is greater than dose 2 implies that the median survival time with dose 2 is greater than that with dose 1. This is represented as, ${\overset{¯}{T}}_{1}=\frac{1}{\theta_{1}}<{\overset{¯}{T}}_{2}=\frac{1}{\theta_{2}}$ and $\overset{˜}{T_{1}}=\frac{\ln(2)}{\theta_{1}}<\overset{˜}{T_{2}}=\frac{\ln(2)}{\theta_{2}}$. Thus, in terms of hypotheses, we can state $H_{0}:\overset{˜}{T_{1}}=\overset{˜}{T_{2}}$ and $H_{1}:\overset{˜}{T_{1}}<\overset{˜}{T_{2}}$. When there is no censoring, the median survival time estimator, denoted as $\overset{ˆ}{T}$, is unbiased.

Next, consider the case with no MPE. The main issue is to determine SS to verify that Pr(${\overset{ˆ}{T}}_{1}<{\overset{ˆ}{T}}_{2})\geq$ *Q* for a given threshold *Q*. In this context, Pr(${\overset{ˆ}{T}}_{1}$ < ${\overset{ˆ}{T}}_{2}$) is $p_{sup}$.

Using MPE the efficacious treatment is chosen in two cases:

(1) The margin is exceeded by the observed difference in mean survival time between the other level and the more effective dose level.
(2) In a situation of practical equivalence with only two possibilities the most efficacious treatment is selected with a probability of 50% provided other factors (like toxicity, cost) are unrelated to efficacy.

Let $\pi_{n}$(MPE) be defined as the likelihood of selecting the more effective treatment given an SS *n* and a given MPE, like three months of survival. Then, for the exponential distribution, we have

(2.1) $$\pi_{n}(MPE)=Pr({\overset{ˆ}{T}}_{1}-{\overset{ˆ}{T}}_{2}<-MPE)+0.5\times Pr(-MPE\leq {\overset{ˆ}{T}}_{1}-{\overset{ˆ}{T}}_{2}<MPE).$$

Here, Pr($T_{1}-T_{2}<$ -MPE) represents $p_{sup}$, denoting the probability of the trial concluding with dose 2 being more effective. Additionally, Pr(-MPE $\leq T_{1}-T_{2}<$ MPE) is $p_{equi}$, signifying the probability of the trial resulting in a state of practical equivalence. The resulting SS, denoted as *n*, is determined to ensure that $\pi_{n}$(MPE) exceeds a specified threshold *Q*.

Consider survival data with right censoring next. In this case, we observe $\left(U_{i},\delta_{i}\right)$, i=1,2,...,n, where $U_{i}$= min$\left(T_{i},C_{i}\right)$, $\delta_{i}$ = I($T_{i}$ ≤ $C_{i}$), and $C_{i}$ representing the random potential censoring time. Since $T_{i}$ is independent of $C_{i}$, we assume non-informative right censoring. Considering that the $\left(U_{i},\delta_{i}\right)$, i=1,2,...,n are exponentially distributed in an identical and independent manner, the likelihood is

(2.2) $$L(\theta )=\prod_{i=1}^{n}{\left(\theta e^{-\theta u_{i}}\right)}^{\delta_{i}}{\left(e^{-\theta u_{i}}\right)}^{1-\delta_{i}}=\theta^{r}e^{-\theta W},$$

where $r=\sum_{i=1}^{n}\delta_{i}$ and $W=\sum_{i=1}^{n}u_{i}$. The first and second derivatives of the log-likelihood can be expressed as $\frac{\partial \ln L(\theta )}{\partial \theta }=\frac{r}{\theta }-W$ and $\frac{\partial^{2}\ln L(\theta )}{\partial^{2}\theta }=\frac{-r}{\theta^{2}}$, respectively. In this context, *r* signifies the number of observed events, and *W* is the cumulative sum of observed event times. The observed information *I(θ)* computed by the negation of the second derivative of the log-likelihood, is given by $\frac{r}{\theta^{2}}$. With *r* following a binomial distribution with a non-censoring probability of *p*, for a SS of *n*, we have I(*θ*) = $\frac{np}{\theta^{2}}$. Utilizing the central limit theorem, the sampling distribution of $\overset{ˆ}{\theta }$ = $\frac{r}{W}$ converges to $\overset{ˆ}{\theta }∼N(\theta ,I^{-1}(\theta ))=N(\theta ,\frac{\theta^{2}}{np})$ as n→∞. By using the delta method, the natural logarithm of $\overset{ˆ}{\theta }$ also follows a normal distribution, $\ln\overset{ˆ}{\theta }∼N(\ln\theta ,\frac{1}{np})$. Since the sampling distribution of $\ln\overset{ˆ}{\theta }$ is normal, the distribution of $\ln{\overset{ˆ}{\theta }}_{2}-\ln{\overset{ˆ}{\theta }}_{1}$ is also normal, $\ln{\overset{ˆ}{\theta }}_{2}-\ln{\overset{ˆ}{\theta }}_{1}∼N(\ln\theta_{2}-\ln\theta_{1},\frac{2}{np})$. Here, the ratio $\frac{\theta_{2}}{\theta_{1}}$ corresponds to the hazard ratio (HR). Thus the expression gets reduced to $\ln{\overset{ˆ}{\theta }}_{2}-\ln{\overset{ˆ}{\theta }}_{1}∼N(\ln(HR),\frac{2}{np})$. These findings enable the calculation of the necessary SS through the standard normal distribution. Assuming $\theta_{1}>\theta_{2}$, equation (2.1) can now be expressed as $\pi_{n}(MPE)=Pr({\overset{ˆ}{\theta }}_{2}-{\overset{ˆ}{\theta }}_{1}<-MPE)+0.5\times Pr(-MPE\leq {\overset{ˆ}{\theta }}_{2}-{\overset{ˆ}{\theta }}_{1}<MPE)$. This is followed by calculating the adequate SS to achieve $\pi_{n}(MPE)$

$$\overset{ˆ}{n}=\overset{ˆ}{n}(MPE,Q)=\min\{n\in N:\pi_{n}(MPE)\geq Q\}$$

### 2.2 SS for Weibull randomized trial

Since the Weibull distribution is a generalization of the exponential distribution and is more flexible in terms of hazard shape, it is also used as a benchmark model in time-to-event studies. Let the time-to-event for the kth arm (k = 1, 2) adheres to the Weibull distribution with shape parameter $\alpha_{k}$ and rate parameter $\theta_{k}$. For t>0, the density function is expressed as

$$g(t)=\alpha_{k}\theta_{k}{\left(\theta_{k}t\right)}^{\alpha_{k}-1}e^{-{\left(\theta_{k}t\right)}^{\alpha_{k}}},(\theta_{k},\alpha_{k})>0.$$

Furthermore, let $\mu_{k}$ represent the median time-to-event in the kth arm. For the Weibull distribution, the median time to an event is $\mu =\theta^{-1}\ln2^{\frac{1}{\alpha }}$. We consider doses 1 and 2 practically equivalent if the difference in medians falls within the predefined MPE, i.e.,

$$|\mu_{2}-\mu_{1}|<MPE.$$

If the median event time for dose 2 surpasses that of dose 1 by a margin greater than the predetermined MPE (Margin of Practical Equivalence), denoted as $\mu_{2}-\mu_{1}>$ MPE, then dose 2 is deemed superior to dose 1. The current emphasis lies in estimating the median event time for each treatment arm, while accounting for non-informative random right censoring. The likelihood function pertinent to this scenario can be formulated as follows:

(2.3) $$L(\alpha ,\theta )=\prod_{i=1}^{n}{\left\{\alpha \theta^{\alpha }u_{i}^{\alpha -1}\right\}}^{\delta_{i}}e^{-{\left(\theta u_{i}\right)}^{\alpha }}$$

Using profile likelihood, the MLE of *θ* [29] (and supplementary text of [17]) is calculated as follows:

(2.4) $$\overset{ˆ}{\theta }(\alpha )={\left(\frac{\sum_{i=1}^{n}u_{i}^{\alpha }}{\sum_{i=1}^{n}\delta_{i}}\right)}^{-1/\alpha }$$

and after obtaining $\overset{ˆ}{\theta }$, the estimate of $\overset{ˆ}{\alpha }$ can be obtained easily [27]. According to the invariance principle, the MLE of the median becomes $\overset{ˆ}{\mu }={\overset{ˆ}{\theta }}^{-1}\ln2^{\frac{1}{\overset{ˆ}{\alpha }}}$. Then, employing the delta method, the standard error (SE) for $\overset{ˆ}{\mu }$ in large samples can be obtained.

To estimate the SS, we use a large sample approximation, wherein ${\overset{ˆ}{\mu }}_{k}∼N(\mu_{k},{\overset{ˆ}{\sigma }}_{\mu ,k}^{2})$, where ${\overset{ˆ}{\mu }}_{k}$ signifies the median time-to-event in treatment arm *k* under the Weibull model, and ${\overset{ˆ}{\sigma }}_{\mu ,k}$ stands for the corresponding standard error (SE). For a pre-specified MPE, the probability of selecting the more effective dosage level is calculated as:

(2.5) $$\pi_{n}(MPE)=Pr({\overset{ˆ}{\mu }}_{2}-{\overset{ˆ}{\mu }}_{1}>MPE)+0.5\times Pr(|{\overset{ˆ}{\mu }}_{2}-{\overset{ˆ}{\mu }}_{1}|\leq MPE)=1-\frac{1}{2}\Phi \{\frac{MPE-(\mu_{2}-\mu_{1})}{\sqrt{{\overset{ˆ}{\sigma }}_{\mu ,1}+{\overset{ˆ}{\sigma }}_{\mu ,2}}}\}-\frac{1}{2}\Phi \{\frac{-MPE-(\mu_{2}-\mu_{1})}{\sqrt{{\overset{ˆ}{\sigma }}_{\mu ,1}+{\overset{ˆ}{\sigma }}_{\mu ,2}}}\}$$

where Φ(.) denotes the cumulative probability of N(0,1). Using $\pi_{n}$(MPE) and ensuring a probability of selection of at least Q, the required SS for identifying the most effective treatment is determined

$$\overset{ˆ}{n}=\overset{ˆ}{n}(MPE,Q)=\min\{n\in N:\pi_{n}(MPE)\geq Q\}$$

To verify the power computations using equation (2.5), simulated Weibull datasets were generated with shape and rate parameters ($\alpha_{1},\theta_{1}$) and ($\alpha_{2},\theta_{2}$) for doses 1 and 2, respectively. For each treatment arm, k∈1,2, the MLE of the median ${\overset{ˆ}{\mu }}_{k}$ and its corresponding standard error $\sigma_{\mu ,k}$ were calculated [30]. The probability of choosing a more efficacious treatment $\pi_{n}(MPE)$ for a specified MPE was calculated using two methods:

(1) Using a large sample approximation as given in equation (2.5).
(2) Empirically by averaging $\pi_{n}(MPE)=Pr({\overset{ˆ}{\mu }}_{2}-{\overset{ˆ}{\mu }}_{1}>MPE)+0.5\times Pr(|{\overset{ˆ}{\mu }}_{2}-{\overset{ˆ}{\mu }}_{1}|\leq MPE)$.

### 2.3 SS for generalized exponential randomized trial

For the generalized exponentially distributed data, our starting point involved instances without censoring, where the time taken by each participant to experience a specific event is documented. As previously, let a trial encompassing two different dosage levels, denoted as levels 1 and 2. The survival times *T* were distributed according to the generalized exponential distribution. More specifically, $T_{1}∼$ GenExp($\alpha_{1},\theta_{1}$) and $T_{2}∼$ GenExp($\alpha_{2},\theta_{2}$), where $\alpha_{i}>0$ and $\theta_{i}>0$ for i = 1,2. The generalized exponential density function is

(2.6) $$g(t)=\alpha \theta e^{-\theta t}{\left\{1-e^{-\theta t}\right\}}^{\alpha -1}$$

For a generalized exponential distribution governed by a shape parameter *α* and a rate parameter *θ*, the median time-to-event is given by $-\frac{1}{\theta }(\ln(1-0.5^{1/\alpha }))$. We deem doses 1 and 2 as practically equivalent if the disparity between their medians falls within the MPE range, expressed as $|\mu_{2}-\mu_{1}|<MPE$. When the median of dose 2 surpasses dose 1 by a margin greater than the MPE, which is expressed as $\mu_{2}-\mu_{1}>$ MPE, we consider dose 2 to be superior to dose 1. Moving forward, our objective is to estimate the median event time for each of the two treatment arms by utilizing non-informative random right censoring. In the case of a particular arm, the data is represented as tuples $\left(U_{i},\delta_{i}\right)$, where $U_{i}=\min(T_{i},C_{i})$, and $\delta_{i}$ is one if $T_{i}\leq C_{i}$ and zero otherwise. The likelihood is

(2.7) $$L(\alpha ,\theta )=\prod_{i=1}^{n}\alpha \theta e^{-\theta t}{\left\{1-e^{-\theta t}\right\}}^{\alpha -1}$$

Using the MLE method, we estimated the parameters *α* and *θ* while using the invariance property, the MLE of the median is thus obtained, which is $\overset{ˆ}{\mu }=-\frac{1}{\overset{ˆ}{\theta }}(\ln(1-0.5^{1/\overset{ˆ}{\alpha }}))$.

To estimate the SS, a large sample approximation is employed where ${\overset{ˆ}{\mu }}_{k}$ follows a normal distribution $N(\mu_{k},{\overset{ˆ}{\sigma }}_{\mu ,k}^{2})$. Here, ${\overset{ˆ}{\mu }}_{k}$ represents the median time-to-event in treatment arm *k* based on the generalized exponential model, and ${\overset{ˆ}{\sigma }}_{\mu ,k}$ is the associated SE. Given the MPE, we determine the probability of selecting the more effective dosage level using:

(2.8) $$\pi_{n}(MPE)=Pr({\overset{ˆ}{\mu }}_{2}-{\overset{ˆ}{\mu }}_{1}>MPE)+0.5\times Pr(|{\overset{ˆ}{\mu }}_{2}-{\overset{ˆ}{\mu }}_{1}|\leq MPE)=1-\frac{1}{2}\Phi \{\frac{MPE-(\mu_{2}-\mu_{1})}{\sqrt{{\overset{ˆ}{\sigma }}_{\mu ,1}+{\overset{ˆ}{\sigma }}_{\mu ,2}}}\}-\frac{1}{2}\Phi \{\frac{-MPE-(\mu_{2}-\mu_{1})}{\sqrt{{\overset{ˆ}{\sigma }}_{\mu ,1}+{\overset{ˆ}{\sigma }}_{\mu ,2}}}\}$$

where Φ(.) denotes the cumulative distribution function of N(0,1). Using $\pi_{n}$(MPE) and ensuring a probability of selection of at least Q, the required SS for identifying the most effective treatment is determined by

$$\overset{ˆ}{n}=\overset{ˆ}{n}(MPE,Q)=\min\{n\in N:\pi_{n}(MPE)\geq Q\}$$

For verifying the power calculations presented in equation (2.8), a series of simulations were conducted for various sample sizes n∈5,10,...,100. These simulations involved generating datasets with distinct shape and rate parameters ($\alpha_{1},\theta_{1}$) and ($\alpha_{2},\theta_{2}$) for both dosages. For each treatment arm k∈1,2, the MLE of the median ${\overset{ˆ}{\mu }}_{k}$ and the corresponding SE $\sigma_{\mu ,k}$ are calculated. Subsequently, the probability of selecting a more effective treatment, denoted as $\pi_{n}(MPE)$, is computed for a specified MPE using two different methods:

(1) Using a large sample approximation as given in equation (2.8).
(2) Empirically by averaging $\pi_{n}(MPE)=Pr({\overset{ˆ}{\mu }}_{2}-{\overset{ˆ}{\mu }}_{1}>MPE)+0.5\times Pr(|{\overset{ˆ}{\mu }}_{2}-{\overset{ˆ}{\mu }}_{1}|\leq MPE)$.

## 3 Data generation and results

In survival analysis, the data usually consists of two columns, i.e., survival time and censoring status. Data are generated by varying both parameters of the distribution using the library *temporal* as discussed in McCaw [30]. The steps involved in SS calculation are given as follows.

(1) Generate data from a given distribution.
(2) Estimate the parameters of the model using the optimize function of the R package maxLik. In this study, we used two sets of parameters. The first one is $\alpha_{2}=$2.4, 2.5, 2.6, 2.7, 2.8, 2.9, 3.0 and $\theta_{2}=$0.08 and 0.09, whereas the second set of parameters is $\alpha_{2}=$1.5, 1.6, 1.7, 1.8, 1.9, 2.0, 2.1, 2.2, 2.3, 2.4, 2.5, 2.6, 2.7, 2.8, 2.9, 3.0, and $\theta_{2}=$0.10 and 0.11 for the models considered in this study.
(3) Calculate the median and SE of the median using estimated parameters.
(4) Specify the power or the target probability and calculate the probability for the correct selection of treatment that meets the minimum threshold value.
(5) The required value of *n* is the smallest value for which the condition in Step 4 becomes true.

### 3.1 Results and discussion

This section discusses the results computed using different models. We begin with the values of median survival of the two arms. The median survival in arm 1 in the case of Weibull is 8.3 months which occurs when α=2 and θ=0.10. For arm 2, we vary the values of the parameters such that our initial assumption of $\theta_{1}>\theta_{2}$ remains true. We consider different values of the parameters of the second treatment arm such that the median difference between the two arms is up to 4 months. The results for different median differences of the two arms are discussed below.

#### 3.1.1 SS for Weibull distribution

This section presents and discusses the results of SS calculated for randomized trials under the Weibull distribution. The results for different median differences of the two arms in cases with and without censoring are shown and discussed here. Table 1 considers the cases of censoring from 0% to 40%. It shows that for a given value of the rate parameter say 0.09, a smaller SS is required to attain the condition of practical equivalence for increasing values of the shape parameter. To achieve 80% statistical power, the necessary SS required is 71 patients. This calculation takes into consideration the MPE of one month. Consequently, when dealing with smaller median differences, a larger SS is needed to attain 80% power. On the other hand, as the median difference becomes larger, a smaller SS suffices to achieve the same 80% power. When the censoring rate is raised to 10%, and the shape parameter is adjusted while keeping the rate parameter constant, the necessary SS decreases. However, it is important to highlight that, even in this scenario, larger sample sizes are needed compared to those required in the absence of censoring. We attribute this to the loss of information while censoring. For example, for $\alpha_{2}=2.4$ and θ=0.09 a sample of 72 patients is required to attain 80% power. Now in the case of 20% censoring, the SS rises to 75 under the same conditions. As the censoring rate increases from 20% to 30% the SS gets larger, i.e., the SS rises to 81. With 40% censoring, the SS needed is 88. This leads us to the observation that as the rate of censoring increases, a greater SS becomes necessary to achieve the desired power level (80% in our study). Another noteworthy aspect is the median difference's influence. A decrease in median difference demands a larger SS for reaching the desired power, while an increase in median difference demands a smaller SS to achieve the desired power while keeping all other factors constant.

**Table 1:** Sample size for Weibull randomized trial.

censoring (%)	*θ*_2_	$\alpha_{2}$						
		2.4	2.5	2.6	2.7	2.8	2.9	3.0
0	0.08	10	10	10	10^a^	10^a^	10^a^	10^a^
	0.09	71	48	36	29	26	22	20

10	0.08	10	10	10	10^a^	10^a^	10^a^	10^a^
	0.09	72	49	39	31	27	23	20

20	0.08	10	10	10	10^a^	10^a^	10^a^	10^a^
	0.09	75	50	40	32	27	24	20

30	0.08	10	10	10	10^a^	10^a^	10^a^	10^a^
	0.09	81	55	42	35	29	25	22

40	0.08	10	10	10	10^a^	10^a^	10^a^	10^a^
	0.09	88	60	46	38	33	28	25

a Adjusted value.

As in Table 1 one can notice that for θ=0.08 the value for SS is 10 for all values of $\alpha_{2}$. This arises due to a greater difference in the two median arms compared to the other value which is equal to 0.09. When the median difference between the two arms gets greater, a smaller SS is required to achieve the practical equivalence. Therefore, in this case, the SS is smaller as the difference is greater (the minimum value of SS in the result).

**Figure 1:**
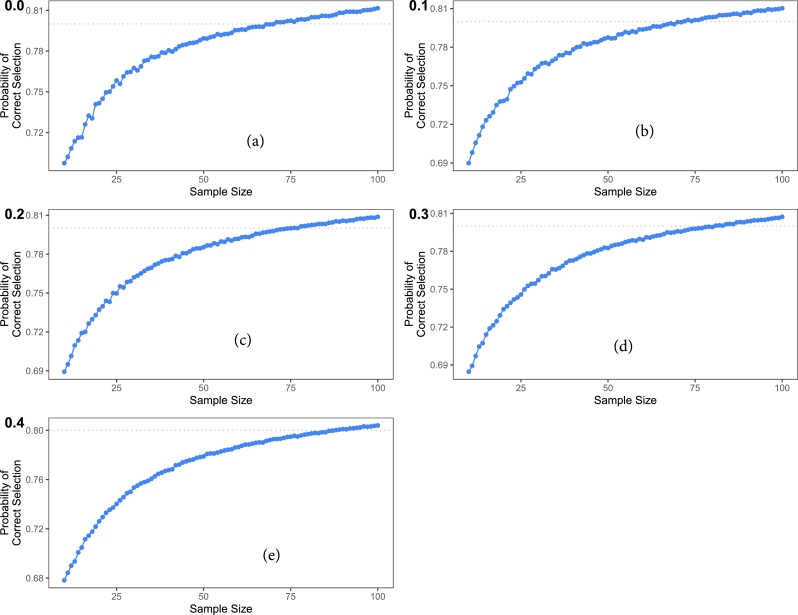
Sample Size for Weibull randomized trials assuming (a) 0%, (b) 10%, (c) 20%, (d) 30%, and (e) 40% censoring.

We also assumed $\theta_{2}=0.10$ and 0.11, where the median survival in arm 1 in the case of Weibull is 6.5 months which occurs when α=1.5 and $\theta_{1}=0.12$. For arm 2, we vary the values of the parameters such that our initial assumption of $\theta_{1}>\theta_{2}$ remains true. We consider different values of the parameters of the second treatment arm such that the median difference between the two arms is up to 4 months. The results for different median differences of the two arms are listed in Table 2. Table 2 considers the cases of censoring from 0% to 40%. It shows that for a given value of the rate parameter say 0.11, a SS of 60 patients is required to attain the condition of practical equivalence for increasing values of the shape parameter. Consequently, when dealing with smaller median differences, a larger SS is needed to attain 80% power. On the other hand, as the median difference becomes larger, a smaller SS suffices to achieve the same 80% power. When the censoring rate is raised to 10%, the necessary SS increases. The results are further illustrated in Fig. 1.

**Table 2:** Sample size for Weibull randomized trial.

censoring (%)	*θ*_2_	$\alpha_{2}$															
		1.5	1.6	1.7	1.8	1.9	2.0	2.1	2.2	2.3	2.4	2.5	2.6	2.7	2.8	2.9	3.0
0	0.10	18	15	13	11	10	10	10	10	10	10	10	10	10	10	10	10
	0.11	61	42	33	28	23	19	17	16	14	13	13	12	11	10	10	10

10	0.10	18	16	14	12	11	10	10	10	10	10	10	10	10	10	10	10
	0.11	62	43	33	28	23	20	19	16	14	13	13	12	12	11	10	10

20	0.10	20	16	14	12	11	10	10	10	10	10	10	10	10	10	10	10
	0.11	66	46	35	30	25	22	19	17	17	14	14	13	12	11	10	10

30	0.10	21	17	15	13	12	11	10	10	10	10	10	10	10	10	10	10
	0.11	69	49	37	31	27	23	21	19	17	15	15	13	12	12	11	10

40	0.10	22	19	15	14	13	12	11	10	10	10	10	10	10	10	10	10
	0.11	76	51	41	33	28	25	22	20	19	16	15	14	13	12	11	10

#### 3.1.2 SS for the generalized exponential distribution

This section presents and discusses the results of different median differences of the two arms in cases with and without censoring for a randomized trial assuming the generalized exponential distribution.

Table 3 lists the results for SS estimation with 0% to 40% censoring. It shows that for a given value of the rate parameter say 0.08 smaller SS is required to attain the condition of practical equivalence for increasing values of the shape parameter. The calculations show that for five-month difference in the median between the two arms, a SS of 15 patients per arm is needed to attain 80% power with the MPE of one month. For the value 0.09 of the rate parameter, the median difference gets smaller for the same values of shape parameters. Hence, as the shape parameter remains constant, the median difference diminishes, and for narrower median differences, a larger SS is necessary to achieve 80% power. Conversely, when the median difference rises, a smaller SS suffices to attain 80% power.

**Table 3:** Sample size for generalized exponential randomized trial.

censoring (%)	*θ*_2_	$\alpha_{2}$						
		2.4	2.5	2.6	2.7	2.8	2.9	3.0
0	0.08	15	13	11	10	10	10^a^	10^a^
	0.09	34	38	22	20	17	15	14

10	0.08	17	14	13	11	11	10	10^a^
	0.09	39	30	25	22	19	17	15

20	0.08	20	16	15	13	11	10	10^a^
	0.09	45	36	29	25	21	19	18

30	0.08	22	19	17	15	14	13	11
	0.09	53	42	34	28	26	23	21

40	0.08	27	23	21	20	16	15	14
	0.09	67	52	41	34	29	27	26

a Adjusted value.

For 10% censoring, the SS increases. However, it is essential to observe that despite this trend, the required SS remains larger for the specific parameter values than that was required in the case without censoring. For example, for $\alpha_{2}=2.4$ and θ=0.09, a sample of 39 patients is required to attain 80% power whereas when there is no censoring, a study would need a SS of 34 patients given the same conditions. Therefore, it can be inferred that the presence of censoring necessitates a larger SS to satisfy the specified conditions. As the censoring rate increases from 10% to 20% understandably the required SS gets larger. The SS which was 34 and 39 in the presence of 0% and 10% censoring, respectively, now rises to 45 under the same conditions. Similarly, as the censoring rate increases from 20% to 30%, the required SS gets greater. The SS which was 45 in the presence of 20% censoring, now rises to 53 under the same conditions. The SS, which is 53 under 30% censoring, grows to 67 for 40% censoring. This illustrates that as the censoring rate becomes greater, a larger SS becomes necessary to achieve the designated power of 80%. Similarly, the results for other censoring rates can be interpreted.

**Figure 2:**
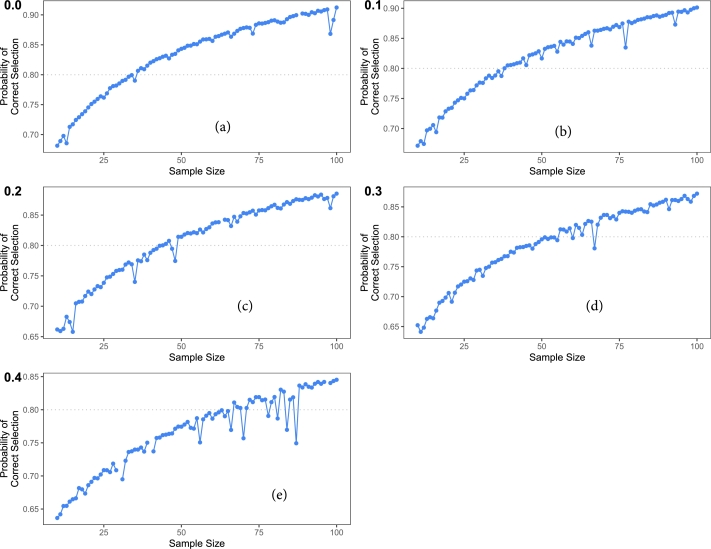
Sample Size for generalized exponential randomized trials assuming (a) 0%, (b) 10%, (c) 20%, (d) 30%, and (e) 40% censoring.

Table 4 considers the case with 0% to 40% censoring. It shows that for a given value of the rate parameter say 0.11 smaller SS is required to attain the condition of practical equivalence for increasing values of the shape parameter. Calculations show that for five months difference in median between the two arms SS of 35 patients per arm is needed to attain 80% power. For the value 0.11 of the rate parameter, the median difference gets smaller for the same values of shape parameters. Hence, as the shape parameter remains constant, the median difference diminishes, and for narrower median differences, a larger SS is necessary to achieve 80% power. Conversely, when the median difference rises, a smaller SS suffices to attain 80% power. The SS increases as the censoring rate increases. For example, the SS which was 28 and 30 in the presence of 0% and 10% censoring for $\alpha_{2}=1.6$, respectively, now rises to 36 for 20% censoring. The results are further illustrated in Fig. 2.

**Table 4:** Sample size for generalized exponential randomized trial.

censoring (%)	*θ*_2_	$\alpha_{2}$															
		1.5	1.6	1.7	1.8	1.9	2.0	2.1	2.2	2.3	2.4	2.5	2.6	2.7	2.8	2.9	3.0
0	0.10	35	28	20	18	15	14	12	11	10	10	10	10	10	10	10	10
	0.11	47	32	25	23	20	18	15	14	12	11	10	10	10	10	10	10

10	0.10	39	30	24	20	17	15	13	12	12	10	10	10	10	10	10	10
	0.11	52	43	34	28	24	19	17	16	14	13	12	11	11	10	10	10

20	0.10	43	36	27	25	21	17	15	13	12	11	10	10	10	10	10	10
	0.11	60	45	40	31	25	21	19	17	16	14	13	12	11	10	10	10

30	0.10	50	40	28	26	23	21	17	16	14	13	12	11	11	10	10	10
	0.11	72	51	40	34	31	27	23	20	18	16	15	14	13	12	11	10

40	0.10	60	43	34	31	28	25	21	20	18	15	14	13	13	12	11	10
	0.11	80	56	46	37	33	30	26	23	22	20	19	17	16	15	14	10

### 3.2 Comparison of results of the two distributions

After calculating the SS assuming Weibull and generalized exponential distributions, the question now arises which distribution is better to determine SS. The better one under the same conditions would be the one that requires a smaller SS compared to the other. As one can notice from the tables the SS required for generalized exponential distribution is smaller compared to the Weibull distribution for the given value of shape and rate parameter. For example, assuming $\alpha_{2}=2.4$ and $\theta_{1}=0.09$ to attain 80% power without censoring, the Weibull distribution requires a SS of 71, while the generalized exponential distribution requires a SS of 34. However, as the censoring rate increases, the SS also increases but it is smaller for the generalized exponential distribution compared to the Weibull distribution. Similarly, for *α* = 1.5 and *θ* = 0.11, to attain 80% power, the Weibull distribution gave a SS of 60, while the generalized exponential distribution requires a SS of 28 without censoring. This demonstrates that the generalized exponential distribution outperforms the Weibull distribution in achieving the stated practical equivalence under the MPE of one month and 80% power. An important observation here pertains to the value of the MPE. When the MPE value is zero, the probability of selection of the right treatment approaches one as the SS increases. Conversely, if the MPE is greater, i.e., 2, which indicates that the expected median difference is within the MPE, the probability of selection of treatment approaches 0.5 as the SS increases. Generally, when the anticipated difference in the median is within the MPE, the probability of selecting the more effective treatment is expected to decrease towards 0.5 with larger sample sizes. This phenomenon is due to the increasing likelihood that the median difference will fall within the MPE range as the SS grows. In the case of small sample sizes, the more effective treatment could inadvertently demonstrate a superiority over the competitor by a margin exceeding the MPE. It is important to emphasize that planning a trial holds limited practical significance in cases where the difference in medians is within the MPE. For this reason, an MPE of one is chosen.

## 4 Conclusion and recommendations

The goal of randomized selection trials is to suggest the best treatment option. However, the treatment decision must take into account more than just efficacy unless there is a considerable difference in the options' efficacy. The MPE can be incorporated into the research design phase to achieve the goal of best treatment systematically. Randomized trials are particularly relevant when a recognized standard of care is absent. In such cases, the notion of Type I error does not directly relate to sample size calculation, as the absence of an internal competitor. However, researchers can define a minimal efficacy threshold that a treatment approach should satisfy to warrant further consideration. Considering external or historical references, such a threshold can be created.

This study focused on determining the necessary sample size for a selection randomized trial involving time-to-event endpoints, utilizing parametric survival distributions. Our methodology is particularly suitable when there's limited prior knowledge about the survival curve or when external data indicates that Weibull or generalized exponential models suitably approximate the curves. While the exponential model assumes a constant hazard function, the Weibull and generalized exponential models exhibit a monotonic hazard function. In cases where there is a possibility of a different hazard function shape, simulations can be employed to derive the requisite sample size. In this research, a probability threshold of 0.80 is taken. The treatment's probability of being correctly selected is considered higher than this threshold value for a specific sample size *n*. The minimum value of *n* for which it attains the threshold value of 0.80 is thus the required sample size. It is shown that a large sample size is required if there is a large censoring and vice versa. Similarly, assuming the same values of parameters, generalized exponential results into smaller sample sizes to achieve the same power.

In the future, this study can be expanded by employing other survival distributions such as gamma, generalized gamma, and log-normal distributions.

## Computational code availability

The R codes used in this study is available from the corresponding author.

## Funding

The authors received no specific funding for the article.

## Ethics approval and consent to participate

Not Applicable.

## CRediT authorship contribution statement

**Muhammad Hamza Akbar:** Writing – original draft, Software, Investigation. **Sajid Ali:** Visualization, Project administration, Formal analysis, Conceptualization. **Ismail Shah:** Writing – original draft, Supervision, Resources, Investigation. **Hana N. Alqifari:** Writing – original draft, Validation.

## Declaration of Competing Interest

The authors declare that they have no known competing financial interests or personal relationships that could have appeared to influence the work reported in this paper.

## Data Availability

Data sharing is not applicable to this article as no new data were created or analyzed in this study.
